# Kalium 2.0, a comprehensive database of polypeptide ligands of potassium channels

**DOI:** 10.1038/s41597-019-0074-x

**Published:** 2019-05-27

**Authors:** Valentin M. Tabakmakher, Nikolay A. Krylov, Alexey I. Kuzmenkov, Roman G. Efremov, Alexander A. Vassilevski

**Affiliations:** 1 Shemyakin-Ovchinnikov Institute of Bioorganic Chemistry, Russian Academy of Sciences, Moscow, 117997 Russia; 2 School of Biomedicine, Far Eastern Federal University, Vladivostok, 690950 Russia; 3 National Research University Higher School of Economics, Moscow, 101000 Russia; 4 Moscow Institute of Physics and Technology (State University), Dolgoprudny, Moscow Oblast 141700 Russia

**Keywords:** Ion channels in the nervous system, Potassium channels, Receptor pharmacology, Protein databases

## Abstract

Potassium channels are the most diverse group of ion channels in humans. They take vital parts in numerous physiological processes and their malfunction gives rise to a range of pathologies. In addition to small molecules, there is a wide selection of several hundred polypeptide ligands binding to potassium channels, the majority of which have been isolated from animal venoms. Until recently, only scorpion toxins received focused attention being systematically assembled in the manually curated Kalium database, but there is a diversity of well-characterized potassium channel ligands originating from other sources. To address this issue, here we present the updated and improved Kalium 2.0 that covers virtually all known polypeptide ligands of potassium channels and reviews all available pharmacological data. In addition to an expansion, we have introduced several new features to the database including posttranslational modification annotation, indication of ligand mode of action, BLAST search, and possibility of data export.

## Abstract (summary)

**Table:** 

Design Type(s)	database creation objective • data integration objective
Measurement Type(s)	ligand
Technology Type(s)	digital curation
Factor Type(s)	Species
Sample Characteristic(s)	Serpentes • Scorpiones • Araneae • Anemone • Conus <genus> • Ancylostoma ceylanicum • Anoplius samariensis • Apis mellifera • Brugia malayi • Heloderma horridum horridum • Homo sapiens • Pseudoplectania nigrella • Scolopendra mutilans • Scolopendra subspinipes dehaani

Machine-accessible metadata file describing the reported data (ISA-Tab format)

## Background & Summary

Potassium (K^+^) channels are a superfamily of integral membrane proteins responsible for selective potassium ion permeation through cell membranes. Activity of K^+^ channels regulates cell excitability and controls the shape of the action potential^1^. Being present in various cells they participate in processes as diverse as cognition, muscle contraction, and hormone secretion^2^. K^+^ channels are composed of two or four major α subunits that form the pore and auxiliary β subunits^3,4^. K^+^ channels of mammals are classified into four groups according to gene homology and structure of the α subunits: calcium- and sodium-activated (K_Ca_ and K_Na_), inwardly rectifying (K_ir_), two pore domain (K_2P_), and voltage-gated (K_V_) potassium channels^5–9^.

A large number of various molecules can interact with K^+^ channels. Three major classes are often cited: metal ions, low-molecular-mass substances, and polypeptides^10^. Despite structural differences most K^+^ channel ligands may either physically occlude the channel pore, or change channel properties through gating modification^11^. Polypeptide ligands are of special interest to researchers due to high affinity (often active at nanomolar or even subnanomolar concentrations) and selectivity towards their targets. Most of these molecules are toxins from venomous animals but some are found in different sources^12–15^. Polypeptide ligands play a key role in unravelling the functions of K^+^ channels and serve a pool of natural prototypes for drug discovery^16^.

>95% of K^+^ channel polypeptide ligands have been identified in just five groups of organisms^10^ and scorpion toxins (KTx) provide >50% of this variability. They consist of ∼20–75 amino acid residues and usually contain 2–4 disulfide bridges^17^. Five structural folds are described for KTx: cysteine-stabilized α-helix/β-sheet (CSα/β), cysteine-stabilized helix-loop-helix (CSα/α) with two or three disulfide bonds, Kunitz-type, and inhibitor cystine knot (ICK) folds^18^. KTx generally inhibit K_V_ and K_Ca_ channels through pore blockage^10^. The most famous ligands of K^+^ channels from snakes are dendrotoxins that contain ∼55–60 amino acid residues and form a Kunitz-type fold^19,20^. Another important group is myotoxin-like polypeptides composed of ∼40–50 amino acids, which assume a similar fold to human β-defensins and display versatile activities including K_V_ channel inhibition^21^. Spider toxins containing ∼30–40 amino acid residues and forming the ICK motif inhibit mostly K^+^ channel activation via interactions with the voltage sensor^22^. The founding member of this group is hanatoxin^23^ and their peculiar ability is to partition into membranes and interact with the channels by lateral association within the membranes^22,24^. Some weak pore blockers of K_V_ channels assuming the Kunitz-type fold have also been found in spider venom^25^. K^+^ channel ligands from sea anemones are composed of ∼35–65 amino acid residues and can be subdivided into three subgroups by structural features^26^. Their spatial structures are presented by a combination of α and/or 3_10_-helices, several β-strands, or the Kunitz-type fold^18,26^. Sea anemone toxins often bear posttranslational modifications and inhibit K_V_ and K_Ca_ channels^10^. Cone snails use a number of different structural classes of toxin to target K_V_ channels: κA-, κO-, κM-, κI-, κJ-, and κL-conotoxins^27,28^. These polypeptides comprise ∼20–30 amino acid residues and present diverse disulfide patterns and folds^29^. Two toxins have a particularly unusual structure: conkunitzin-S1, a 60 residues-long polypeptide with the Kunitz-type fold^30^, and contryphan-Vn of just nine amino acids^31^. Conotoxins are also often subjected to posttranslational modifications. In addition, a comparatively small number of molecules affecting K^+^ channels has been found in some species of bees, worms, lizards, fungi, and scolopendra^13,14,32–34^; moreover, human β-defensin 4A displays activity against several K_V_ isoforms^15^.

The first version of Kalium comprised only scorpion toxins^35^, while its current expansion and update includes all known polypeptide ligands identified in living organisms. For all these compounds detailed activity data are provided collected from original manuscripts. Several major improvements have been introduced, such as the indication of toxin mode of action, BLAST search, and possibility to export data in .csv (comma-separated) or .txt (tab-delimited) format. Kalium is manually curated, and presents a comprehensive list of all known polypeptide K^+^ channel ligands available to users. Kalium is of primary utility to researchers investigating the structure and function of K^+^ channels, toxinologists addressing the variability and mode of action of natural toxins, pharmacologists and research and development managers involved in drug discovery targeting K^+^ channels, and biochemical community in general.

## Methods

### Data sources and curation

Data for Kalium 2.0 were assembled from scorpion venom peptide entries already present in the first release of Kalium^35^, which was updated and expanded by adding the available information on K^+^ channel ligands from other organisms. As a result, Kalium 2.0 contains twice as many entries as Kalium 1.0. The compiled data on all publically available sequences of polypeptide ligands of K^+^ channels were obtained from UniProt (http://www.uniprot.org/)^36^. Available PDB structures with links to the RCSB Protein Data Bank (https://www.rcsb.org)^37^ and location of disulfide bonds were also extracted from UniProt. The data set was then manually filtered and refined, including the following steps: removal of peptides with partial sequence, removal of entries supported by genomic or transcriptomic information only, and sorting by the source organism into six groups: snakes, scorpions, spiders, sea anemones, cone snails, and miscellaneous. Kalium 1.0 and 2.0 entries statistics is summarized in Table 1.

**Table 1:** Kalium entries statistics.

Source organisms		Entries added		Current number of entries
		Kalium 1.0	Kalium 2.0	
Scorpions		174	19	193
Snakes		—	29	29
Spiders		—	50	50
Sea anemones		—	35	35
Cone snails		—	19	19
Miscellaneous	Nematodes	—	2	2
	Hymenopterans	—	4	4
	Lizards	—	1	1
	Human	—	1	1
	Fungi	—	1	1
	Centipedes	—	5	5
Total		174	164	340

Partially sequenced polypeptides were excluded because they cannot be used straightforwardly for nomenclature or in further research and bring confusion to the entire data set. Sequences obtained from transcriptomes without verification on protein level were also left out because (i) they are of less interest for researchers, (ii) there is differential presence or absence of transcriptomic entries from different organisms in UniProt-supported toxin classification and (iii) transcriptomic studies grow fast in numbers and often provide data of low accuracy.

In many cases, experimentally measured molecular masses for natural polypeptides are unavailable. For this reason, molecular masses were calculated for every curated Kalium 2.0 entry. Commonly, the task of precise molecular mass calculation is more complicated than it seems to be, due to co- and posttranslational modifications. In addition to the more widespread cleavage of signal and propeptides, N-terminal cyclization of glutamine, C-terminal amidation, and disulfide bridge formation, as an improvement in Kalium 2.0 we also took into consideration the following modifications: N^ε^-formylation of lysine, γ-carboxylation of glutamic acid, and γ-hydroxylation of proline. Tables of amino acid masses and modifications from the FindMod tool of the ExPASy server^38,39^ were used for calculations:

https://web.expasy.org/findmod/findmod_masses.html#aas — amino acid molecular masses,

https://web.expasy.org/findmod/PYRRE.html — cyclization of N-terminal glutamine into pyroglutamate,

https://web.expasy.org/findmod/AMID.html — amidation of C-terminal amino acids,

https://web.expasy.org/findmod/FORM.html — N^ε^-formylation of lysine,

https://web.expasy.org/findmod/GGLU.html — γ-carboxylation of glutamic acid,

https://web.expasy.org/findmod/HYDR.html — γ-hydroxylation of proline.

Disulfide bonds were taken into account by subtracting two hydrogen atomic masses from the mass of two cysteines. Molecular masses for O-glycosylated polypeptides were calculated only for the aglycone (polypeptide) parts. Table 2 shows good accordance of calculated and measured molecular masses for several Kalium entries.

**Table 2:** Comparison of ligand molecular masses measured experimentally and calculated in Kalium.

UniProt ID	Experimental mass, Da	Kalium calculated mass, Da	Known modifications
Q9U3Z3	3569	3571.74	Signal and propeptide cleavage, 4 disulfide bridges, 4 γ-carboxyglutamates, 1 γ-hydroxyproline, C-terminal amidation
P0CG45	2805.84	2807.25	3 disulfide bridges, 7 γ-hydroxyprolines
Q86QT3	4730.8	4730.49	Signal peptide cleavage, 4 disulfide bridges
P84704	2872.5	2873.32	2 disulfide bridges
P0C166	4082.8	4081.99	N-Terminal cyclization of glutamine, C-terminal amidation, 4 disulfide bridges

**Fig. 1:**
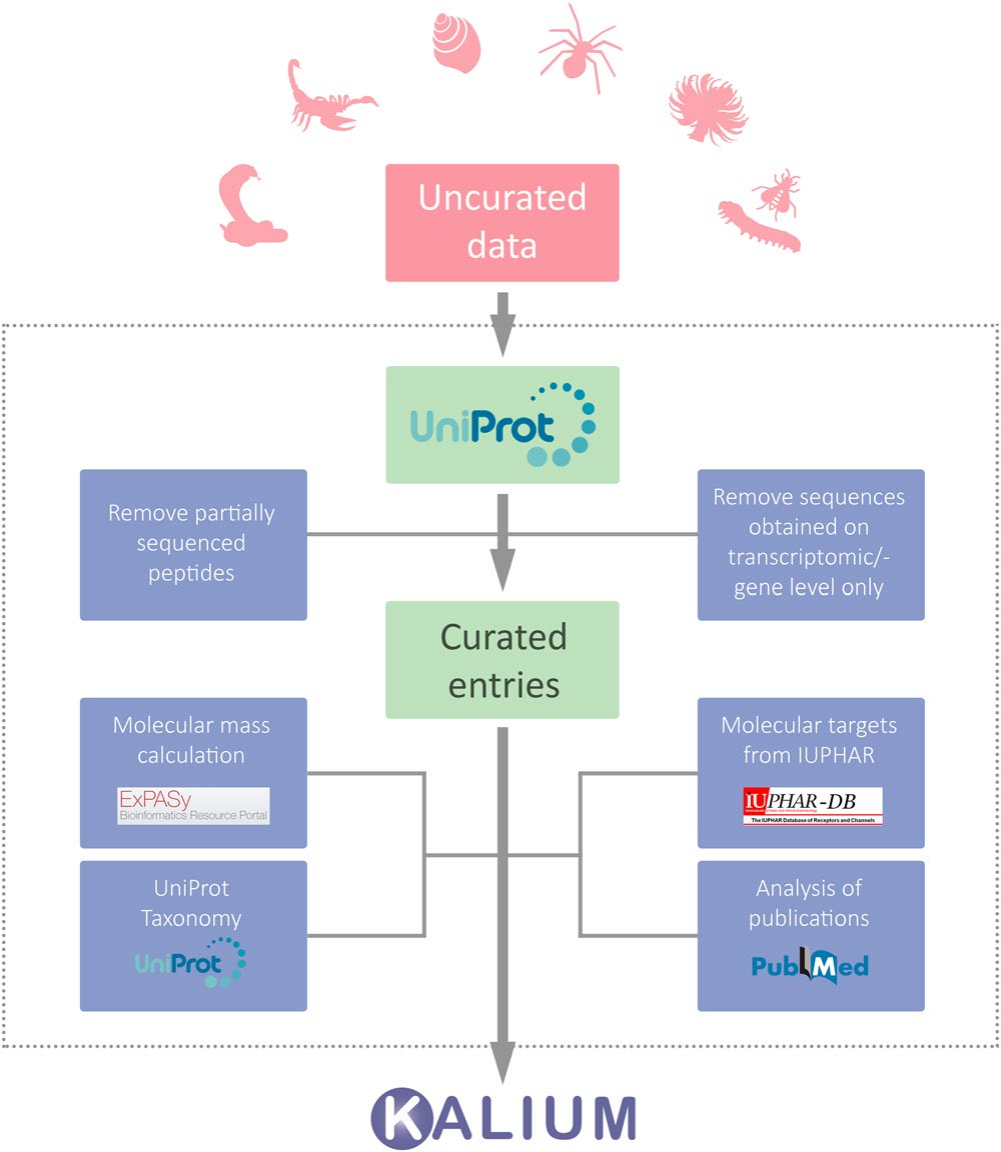
Data sources and curation. Schematic representation of the data stream and curation process in Kalium 2.0.

Further, the Latin name of every source organism was linked to a valid species entry in the UniProt Taxonomy database (UniProt equivalent of NCBI Taxonomy Browser; http://www.uniprot.org/taxonomy/). Comprehensive activity data were added manually from literature and linked to corresponding references in PubMed (https://www.ncbi.nlm.nih.gov/pubmed/) or DOI. Molecular target nomenclature was adopted as recommended by the International Union of Basic and Clinical Pharmacology (IUPHAR; http://www.guidetopharmacology.org), where it was possible (see “Ligand card”). The data stream and curation process are presented in Fig. 1.

### Implementation

Interface to the Kalium database is centered around the main table with data on K^+^ channel ligands, initially sorted according to source organism group, organism name and polypeptide family name or common name. The table supports searching, multi-column ordering and filtering, and multi-row selection. BLAST search and sequence alignment using the Clustal Omega program via UniProt web server is implemented, as well as data export for toxins selected by users; all these options are new in Kalium 2.0. Extended information including detailed activity data (the “Ligand card”) is available for each entry as a special popup window.

Kalium is an OpenUI 5 Model-View-Controller web application built upon a Django web framework and SQLite3 database engine. The web interface consists of single dynamically generated HTML5 page with JSON data being fetched from the server asynchronously via AJAX requests. Standard Django web admin interface is used for data access and curation. Modern HTML5-capable browsers (desktop and mobile variants) are supported.

## Data Records

Original Kalium 1.0 was assembled as a database of K^+^ channel toxins from scorpion venom^35^. Due to database expansion following the addition of K^+^ channel ligands from other organism sources, the structure of Kalium 2.0 was improved. A copy of Kalium database in CSV format can be accessed at Figshare^40^.

### The main window

**Fig. 2:**
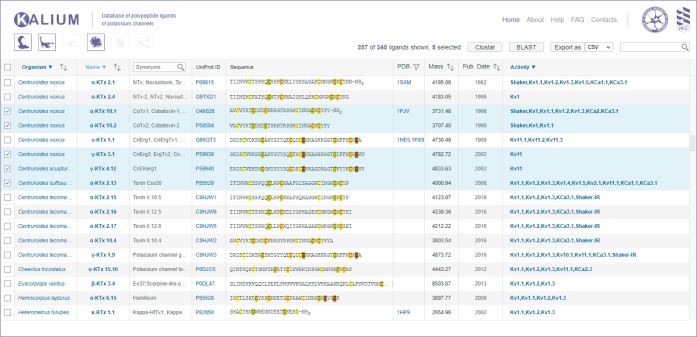
The main window of Kalium 2.0. Top panel consists of the database logo (implementing the home button function), and links to “Home”, “About”, “Help”, “FAQ”, and “Contacts” pages. The second panel contains the organism selection buttons, an indicator of shown and selected entries number, the “Clustal”, “BLAST”, and “Export as” buttons, and a search bar. The main body of the database is presented by a table consisting of fields described in “Data Records”. The figure displays results for the following query: show entries, in which source organisms are snakes, spiders, or sea anemones.

The main window of Kalium is presented by one large general table, in which all data about K^+^ channel ligands from various sources are assembled (Fig. 2 and Table 3). “Home”, “About”, “Help”, “FAQ”, and “Contacts” located in the top right corner link to pages that contain information about developers and tips. Below those links come buttons “Clustal”, “BLAST”, and “Export as” (a drop-down list of export file format), and a search field. Buttons for source organism selection are located under the Kalium logo in the top left corner. Other control elements of the table are placed in the headers and function to filter information of interest as discussed below. Multiparameter filtering is now an available option in Kalium 2.0.

### Ligand card

**Fig. 3:**
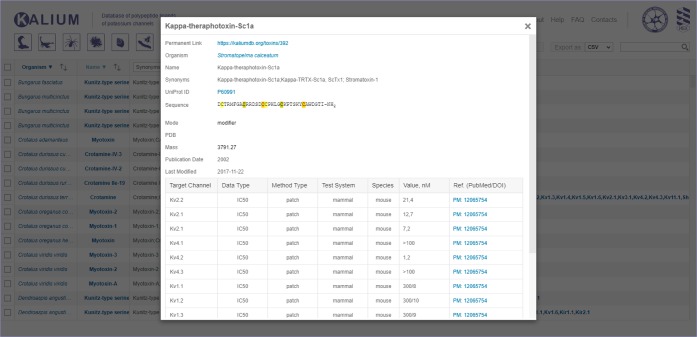
Ligand card overview. Kappa-theraphotoxin-Sc1a is taken as an example. All information present in the general table is duplicated here with certain additions (as described in “Ligand card”). Activity data are summarized in a table located to the bottom of the card.

For each polypeptide entry, detailed information is summarized in the “Ligand card” (Fig. 3) available by clicking on polypeptide name in the field “Name” of the general table. As it was implemented in the first Kalium release, all information presented in the general table is duplicated in the Ligand card in an expanded way^35^. All records of the renewed Ligand card are explained in Tables 3 and 4.

**Table 3:** Description of Kalium 2.0 main window general table.

Table field	Definition
Organism	The Latin name of the source organism.
Name	The nomenclature name or conventionally used name of polypeptide.
Synonyms	Trivial name(s) of polypeptide.
UniProt ID	Unique UniProt ID of polypeptide.
Sequence	Amino acid sequences of mature polypeptides presented in the one-letter code. “–NH_2_” indicates amidation of the C-terminal amino acid; “Z” is for the N-terminal pyroglutamic acid; “O” for 4-hydroxyproline; “**E**” for 4-carboxyglutamic acid; “**K**” for N^6^-formyllysine; “**T**, **S**” are for O-glycosylated threonine and serine; and “**W**” is for D-tryptophan. Cysteine residues are marked; different colors indicate the disulfide bond connectivity.
PDB	Available PDB ID(s) of polypeptide.
Mass	Molecular mass of mature polypeptide calculated taking into account the post-translational modifications. Molecular masses for O-glycosylated polypeptides are marked with the “+” symbol.
Publication date	The date when the polypeptide sequence was first published.
Activity	The list of all targets on which the polypeptide was ever tested.

**Table 4:** Description of Ligand card records.

Record	Definition
Permanent link	Unique link for the Ligand card which can be used for citation purposes.
Raw sequence	Polypeptide precursor sequence (if available).
Last modified	The date of the latest modification to the entry.
Mode	The mode of ligand action on K^+^ channels: *blocker*, blocks ion current by “plugging” the channel pore; *modifier*, alters channel gating and decreases ion currents by voltage-sensor trapping through binding to extracellular receptor sites; *activator*, increases ion currents; and *undefined*.
Target channel	K^+^ channels that were used for toxin activity measurements named according to IUPHAR, except the following: *K*_*x*_*a.b/K*_*x*_*c.d* — heteromeric channel; *Shaker, Shab* — channels from the fruit fly *Drosophila melanogaster*; *Shaker-IR* — Shaker channel with fast N-type inactivation gate removed; *KscA-Shaker* — chimera of prokaryotic channel KscA from the soil bacterium *Streptomyces lividans* and Shaker; *KvAP* — channel from the archaeon *Aeropyrum pernix*; *TSha1* — Shaker-related K^+^ channel from the trout *Oncorhychus mykiss*.
Data type	The type of data reported: dissociation constant (*K*_*d*_), inhibition constant (*K*_*i*_), half-maximal inhibitory concentration (*IC*_*50*_), or half-maximal effective concentration (*EC*_*50*_).
Method type	The experimental method applied: *radio*, radioligand-binding assay; *flux*, rubidium/thallium efflux assay; *patch*, electrophysiology using the patch clamp technique; *volt*, electrophysiology using the voltage clamp technique.
Test system	The cell type used for channel expression: *insect*, *Xenopus oocyte*, *mammalian*, or *snail* (neurons of the mollusk *Helix pomatia*).
Species	The origin organism of the ion channel that was used for measurements. The most common channels belong to *fly*, *rat*, *mouse*, and *human* organisms. Blank means that the origin of ion channel was not specified in the publication.
Value, nM	Numeric value of polypeptide activity (K_d_, K_i_, IC_50_ or EC_50_) presented in nM. These data are collected manually from literature. Values are shown in the following formats: *X* — K_d_, K_i_, IC_50_, or EC_50_ value in nM; ∼*X* — approximate K_d_, K_i_, IC_50_, or EC_50_ value in nM; ≥*X* — ligand had no effect at up to *X* value; *X*/*Y* — means that ligand at concentration *X* reduced ion current through the channels by *Y* percent.
Ref. (PubMed/DOI)	PubMed ID or DOI of the reference article.

Those records that duplicate information of the main window general table are described in Table 3.

### Export file format

Downloadable text file containing data on Kalium entries is generated in the column-separated (default name is “export.csv”) or tabulation-separated (“export.txt”) format. For multiple selected entries, the file consists of truncated Ligand cards appended one by one. Each truncated Ligand card includes UniProt ID, sequence, list of PDB IDs (if available), molecular mass, and mode of action followed by a table of experimentally determined activity data (if available).

## Technical Validation

Database generation process consisted of fetching, filtering and merging manually collected data from the literature and information from the UniProt^36^. UniProt data validation was not performed, since it is one of the most accurately curated biological resources. The records included in Kalium 2.0 are based on published material in peer-reviewed scientific journals; each specific data value is supported by the original references, so users can evaluate the validity and accuracy of the original source. The overall correctness of the database generation process was verified manually. Mass calculation for mature toxins containing 20 common amino acids and modified residues, was checked against the ExPASy server^38,39^.

## Usage Notes

Kalium 2.0 is freely available for users. Most of the original Kalium 1.0 features were upgraded and new features were implemented, we therefore describe all of them in detail below. Moreover, here we give an example of how Kalium 2.0 can be utilized by researchers with specific needs.

### Organism selection buttons

A major new feature of Kalium 2.0 is buttons for organism group selection (Fig. 2). Clicking one or several buttons allows filtering data in the main table according to the source organism groups: snakes, scorpions, spiders, sea anemones, cone snails, and miscellaneous. The “Miscellaneous” group includes K^+^ channel ligands from fungi, worms, bees, wasps, centipedes, lizards, and humans.

### Selecting and manipulating data: Clustal, BLAST, and Export

Check boxes on the left side of the general table permit selection of one or more entries; for all entries selection, users may click once on the column header. Multiple (two or more) entries selection allows performing Clustal alignment request. New features of Kalium 2.0 include an easy BLAST search for multiple sequences and data export for selected polypeptides in a text file.

To submit an alignment request, after entry selection, users need to click the “Clustal” button; the results of Clustal Omega pair/multiple sequence alignment will appear in a new browser tab. Similarly, to submit a BLAST search request, users are required to click the “BLAST” button; the results will appear in separate browser tab for each selected entry. To export data, users are advised to choose the file format (CSV or TXT) in the drop-down list and click the “Export as” button; the resulting file containing data from the selected entries will be generated and sent to the user’s browser.

### Organism

The “Organism” header is the control element for filtering and sorting entries by source species names listed according to current biological classification. One click on the column header opens a drop-down menu, where users can choose one or more species to filter the full data set. The Latin names in the table body are linked to the UniProt Taxonomy database ensuring valid classification.

### Name

The “Name” header is the control element for filtering and sorting entries by polypeptide families and subfamilies according to current nomenclature. As of February 2019, the filtering option is active for families of scorpion toxins only, since the nomenclature of just these molecules is the most conventional, clear and universally recognized (an updated Tytgat-Possani nomenclature^17,41^). “Name” enables selecting toxin family from a drop-down menu. Ligand card opens when clicked on toxin name in the table body.

### Synonyms

The “Synonyms” header is the control element for searching/filtering trivial names of polypeptides. Many scientists identify certain molecules using trivial names only; therefore their inclusion in Kalium 2.0 is a necessity.

### UniProt ID

Click on UniProt ID switches to corresponding UniProt pages.

### PDB

The “PDB” header is the control element for filtering entries by PDB ID (if available). Clicking this filter button will show entries with resolved spatial structure only. All PDB IDs are linked to corresponding Protein Data Bank^37^ pages.

### Mass

The “Mass” header is the control element for sorting entries according to molecular mass. One click on this button will sort entries by ascending order of masses, next click — by descending order.

### Publication date

The “Publication date” header is the control element for sorting entries according to the date when the sequence was first published.

### Activity

The “Activity” header is the control element for filtering and sorting entries by information about activities on different K^+^ channels. One click on the column header opens a drop-down menu, where users can select one or more channels. The header is used to sort entries according to specific targets. Ligand card can be opened for detailed information by clicking on a channel name.

### Ligand card

For user convenience the information of the records “Organism”, “UniProt ID”, “PDB”, and “Ref. (PubMed/DOI)” is linked to corresponding web pages.

### Kalium 2.0 application example

Kalium provides convenient tools to analyze the selectivity features of K^+^ channel ligands. For instance, Kalium may help infer the molecular determinants underlying ligand specificity against particular channel isoforms. Investigators can identify all known polypeptides that were tested against chosen K^+^ channel isoforms by selecting the appropriate channels in the “Activity” header. The most suitable entries may be selected and analyzed by Clustal or BLAST. As a result, assumptions may be made on potentially important residues^42,43^ and this information may be further used to produce artificial molecules with enhanced selectivity or affinity. To perform such analysis without using Kalium is difficult, because it is associated with deep literature search. This search has already been performed during data assembly and is central to manual data curation at Kalium.

## ISA-Tab metadata file

Download metadata file

## Data Availability

Code is available upon request.
